# Antitumor and antiangiogenic effect of the dual EGFR and HER-2 tyrosine kinase inhibitor lapatinib in a lung cancer model

**DOI:** 10.1186/1471-2407-10-188

**Published:** 2010-05-11

**Authors:** Roque Diaz, Paul A Nguewa, Ricardo Parrondo, Carlos Perez-Stable, Irene Manrique, Miriam Redrado, Raul Catena, Maria Collantes, Ivan Peñuelas, Juan Antonio Díaz-González, Alfonso Calvo

**Affiliations:** 1 Division of Oncology, Center for Applied Medical Research (CIMA). University of Navarra, Pamplona, Spain; 2 Geriatric Research, Education, and Clinical Center and Research Service, Veterans Affairs Medical Center; Department of Medicine and Sylvester Comprehensive Cancer Center, University of Miami Miller School of Medicine, Miami Florida 33125, USA; 3 Small Animal Imaging Research Unit, Center for Applied Medical Research (CIMA) and Clínica Universidad de Navarra, Pamplona, Spain; 4 Department of Oncology, Clínica Universidad de Navarra, Pamplona, Spain

## Abstract

**Background:**

There is strong evidence demonstrating that activation of epidermal growth factor receptors (EGFRs) leads to tumor growth, progression, invasion and metastasis. Erlotinib and gefitinib, two EGFR-targeted agents, have been shown to be relevant drugs for lung cancer treatment. Recent studies demonstrate that lapatinib, a dual tyrosine kinase inhibitor of EGFR and HER-2 receptors, is clinically effective against HER-2-overexpressing metastatic breast cancer. In this report, we investigated the activity of lapatinib against non-small cell lung cancer (NSCLC).

**Methods:**

We selected the lung cancer cell line A549, which harbors genomic amplification of EGFR and HER-2. Proliferation, cell cycle analysis, clonogenic assays, and signaling cascade analyses (by western blot) were performed *in vitro*. *In vivo *experiments with A549 cells xenotransplanted into nude mice treated with lapatinib (with or without radiotherapy) were also carried out.

**Results:**

Lapatinib dramatically reduced cell proliferation (*P *< 0.0001), DNA synthesis (*P *< 0.006), and colony formation capacity (*P *< 0.0001) in A549 cells *in vitro*. Furthermore, lapatinib induced G1 cell cycle arrest (*P *< 0.0001) and apoptotic cell death (*P *< 0.0006) and reduced cyclin A and B1 levels, which are regulators of S and G2/M cell cycle stages, respectively. Stimulation of apoptosis in lapatinib-treated A549 cells was correlated with increased cleaved PARP, active caspase-3, and proapoptotic Bak-1 levels, and reduction in the antiapoptic IAP-2 and Bcl-xL protein levels. We also demonstrate that lapatinib altered EGFR/HER-2 signaling pathways reducing p-EGFR, p-HER-2, p-ERK1/2, p-AKT, c-Myc and PCNA levels. *In vivo *experiments revealed that A549 tumor-bearing mice treated with lapatinib had significantly less active tumors (as assessed by PET analysis) (*P *< 0.04) and smaller in size than controls. In addition, tumors from lapatinib-treated mice showed a dramatic reduction in angiogenesis (*P *< 0.0001).

**Conclusion:**

Overall, these data suggest that lapatinib may be a clinically useful agent for the treatment of lung cancer.

## Background

Several targeted therapies are commonly used today as single agents or in combination with radiation or chemotherapeutic drugs for the treatment of solid tumors. Since activation of epidermal growth factor receptor (EGFR) promotes mechanisms leading to tumor growth and progression, EGFR-targeted agents are being widely explored. In addition, some solid tumors, such as lung cancer, exhibit EGFR gene amplification [1,2]. The most clinically advanced EGFR tyrosine kinase inhibitors are erlotinib (Tarceva) and gefitinib (Iressa). Iressa has been shown to be highly effective in non-small-cell lung cancer (NSCLC) patients with activating EGFR mutations [3]. Results with erlotinib in Phase III trials are more promising and the treatment of advanced or metastatic NSCLC with erlotinib is now approved by the FDA [4]. Nevertheless, there remains an urgent need for the identification of additional tyrosine kinase inhibitors that are effective against lung cancer.

Novel drugs such as lapatinib are currently undergoing clinical trials for the treatment of NSCLC, and other tumors [4]. Lapatinib may have a therapeutic advantage over erlotinib because it acts as a dual inhibitor of EGFR (or HER-1) and HER-2 (ErbB2) tyrosine kinases. In lung adenocarcinomas, both EGFR and HER-2 are overexpressed and this is associated with poor prognosis [5]. In addition, previous clinical trials have demonstrated that both EGFR and HER-2 genes are amplified in lung cancer, resulting in the overexpression of these proteins [1,2]. Such overexpression significantly correlates with gene amplification [6]. Studies have shown that EGFR and HER-2 protein overexpression is present in 43-89%, and 30-40% lung cancer specimens, respectively [2]. Therefore, lung tumors with high levels of both EGFR and HER-2 may be appropriate for treatment with lapatinib.

The human NSCLC cell line A549 overexpresses both EGFR and HER-2 and may be an excellent model for testing the efficacy of lapatinib [7,8]. In fact, previous *in vitro *studies have shown that A549 cells are sensitive to this drug [9]. Other lung cancer cells, such as NCI-H358, and Calu3 are also strongly inhibited by lapatinib [9]. In the present work, we analyzed the *in vitro *and *in vivo *efficacy of lapatinib on A549 lung cancer cells. Our results showed that lapatinib decreased cell proliferation and increased apoptosis in these cells *in vitro*. In A549-injected nude mice, treatment with lapatinib significantly reduced tumor activity and angiogenesis. Our data show that lapatinib is an effective drug against NSCLC.

## Methods

### Cell culture

A549 bronchoalveolar carcinoma cells were obtained from the American Type Culture Collection (ATCC, Manassas, VA) and maintained in complete medium, consisting of RPMI 1640 growth medium (Invitrogen/21875-034) with Glutamax^®^, supplemented with 10% heat-inactivated fetal bovine serum (FBS), 1% penicillin-streptomycin (both antibiotics from Invitrogen). Cells were grown at 37°C in a 5% CO_2 _atmosphere. Viable cells were counted in a Neubauer chamber using the Trypan Blue (Sigma-Aldrich, St Louis, MO) exclusion method.

### Cell growth inhibition

Cells were seeded in 96-well plates at a density of 1000 cells/well. After 24 h to allow for attachment, cells were treated with 0.05, 0.5 and 5 μM lapatinib (*Tykerb*^®^, *GlaxoSmithKline*) or left untreated (controls). Cell proliferation was determined with the MTT Cell Proliferation Kit I (Roche, Mannheim, Germany), according to the manufacturer's recommendations. Readings were done at 540/690 nm in the SunRise ELISA plate reader (Tecan Austria GmbH, Salzburg, Austria).

### Clonogenic assay

A549 cells (50,000 per well) were plated (in triplicates) into 6-well plates. After 24 h, cells were treated with 2 μM lapatinib and detached with Trypsin-EDTA (Cambrex Bio Science Verviers, Belgium) one day later. Cells were then counted and 500 cells per 10 cm culture dishes were re-seeded (in triplicates). After 12 days in culture, colonies were fixed with 10% buffered formalin and stained with 2% crystal violet. The number of colonies were determined and normalized to the number of colonies in controls.

### Cell cycle analysis and apoptosis

After incubation with 2 μM lapatinib for 24 h, cells were centrifuged at 1200 rpm for 5 min, fixed in 70% alcohol, kept on ice for 1 h, centrifuged, and washed with PBS. The samples were then resuspended in 500 μL PBS, and 10 μL RNAse A (10 mg/mL) was added and incubated at 37°C for 30 min. After addition of 10 μg/mL propidium iodide (Sigma-Aldrich), the relative DNA content per cell was obtained by measuring the fluorescence of the DNA. The stained cells were detected by flow cytometry using a FACSCalibur (BD Pharmingen, San Diego, CA) and the subsequent analysis was performed with the CELLQuest program. To quantify apoptosis, cells were exposed to 2 or 5 μM lapatinib (for 24 h or 72 h), and active caspase-3 was measured with an apoptosis kit (*FITC Active Caspase-3 Apoptosis Kit*, BD Pharmingen), according to manufacturer's protocol.

### Fluorescence *in situ *hybridization (FISH)

A549 cell suspension was spotted onto a glass slide and air dried. Slides were incubated with protease solution (50 mg/mL pepsin in 0.01 M HCl) at 37°C and fixed with 10% buffered paraformaldehyde. Samples were dehydrated by processing through a series ethanol concentrations. Co-denaturation and hybridization of the probe and cellular DNA were performed with a Hybridizer (DAKO, Glostrup, Denmark), according to the manufacturer's protocol. HER-2/CEP17 FISH probes were obtained from Vysis, Inc. (Dowers Grove, IL). Evaluation of FISH signals was done by counting 100 nuclei and 100 metaphases and calculating the average of HER-2/CEP17 gene copy number per cell.

### Western blot analysis

After treatment with 2 μM laptinib (or PBS as a control) for 72 h, attached and floating A549 cells were collected by centrifugation and lysed at 4°C in lysis buffer (10 mM Tris-HCl, pH 7.4, 1% Triton X-100, 1% Na deoxycholate, 150 mM NaCl, 50 mM NaF, 5 mM EDTA, 0.1% SDS, 1 mM sodium vanadate). Extracts were aliquoted and stored at -80°C for further Western blot analyses. Protein concentrations were determined with the BCA Protein Assay Kit (Pierce, Rockford, IL), resolved by SDS-PAGE, and transferred to polyvinylidene difluoride membranes (Bio-Rad, Hercules, CA). Membranes were blocked with 5% nonfat dry milk in TBS-Tween (1× TBS: 0.05 M Tris-HCl, 0.5 M NaCl, pH 7.36; 0.1% Tween-20) and incubated at the recommended dilution with antibodies specific for PCNA (Dako), GAPDH (AbD Serotec, Kidlington, Oxford, UK), phospho-EGFR, total EGFR, phospho-HER-2, total HER-2, phospho-AKT, total AKT, cleaved PARP (Asp 214), XIAP (all of the latter ones from Cell Signaling Technology, Danvers, MA); c-Myc, cyclin B1, cyclin A, cyclin D1, Mcl-1, IAP-1, IAP-2, survivin (all of these antibodies from Santa Cruz Biotechnology, Santa Cruz, CA); Bcl-xL (BD Pharmingen); and Bak-1 (NT; Upstate, Charlottesville, VA). Membranes were then incubated with the appropriate horseradish peroxidase-conjugated secondary antibody (goat anti-mouse IgG or goat anti-rabbit IgG, Santa Cruz Biotechnology). Immunoblots were developed with the chemiluminescence detection system Lumi-Light PLUS (Roche), exposed to Amersham Hyperfilm™ MP (Amersham, GE Healthcare, Buckinghamshire, UK) and developed with an AGFA automated X-ray film processor.

### A549 xenograft mouse model and treatment with lapatinib

Four week-old male athymic nude (nu/nu) mice (Harlan, Barcelona, Spain) were used in the study and maintained in SPF (Specific Pathogen Free) environment. Animals (n = 5 per group) were inoculated subcutaneously in the left leg (using a sterile 22-gauge needle) with 0.2 mL of Matrigel (BD Pharmingen) containing 1×10^7 ^A549 cells (1:1 volume Matrigel/A549 cells) under ketamine-xylazine anesthesia. Mice were randomized into two groups: a) treated with 100 mg/kg body weight lapatinib (*Tykerb*^®^, *GlaxoSmithKline*) or b) controls (injected with vehicle). Treatments by daily gavage were started one week after cell injection. Tumor width (W) and length (L) were measured once a week with a caliper and the tumor volume (V) was calculated according to the formula: V = 0.5 × W^2 ^× L. All the animal experiments were performed in accordance with the guidelines for the Animal Care Ethics Commission of our institution (University of Navarra) under an approved animal protocol.

### Small animal PET analyses

At the end of treatment (week 4), the effect of lapatinib on tumor activity was measured by positron emission tomography (PET) with the radiotracer 18 fluorodeoxyglucose (^18^F-FDG). Mice were fasted overnight but allowed to drink water *ad libitum*. The following day, mice were anesthetized with 2% isoflurane in 100% O_2 _gas and ^18^F-FDG (10 MBq ± 2 in 80-100 μL) injected via the tail vein. To avoid radiotracer uptake in the hindlimb muscle, ^18^F-FDG uptake was performed under continuous anaesthesia for 50 min. PET imaging was performed in a dedicated small animal Philips Mosaic tomograph (Cleveland, OH), with 2 mm resolution, 11.9 cm axial field of view (FOV) and 12.8 cm transaxial FOV. Anesthetized mice were placed horizontally on the PET scanner bed to perform a static acquisition (sinogram) of 15 min. Images were reconstructed using the 3D Ramla algorithm (a true 3D reconstruction) with 2 iterations and a relaxation parameter of 0.024 into a 128×128 matrix with a 1 mm voxel size applying dead time, decay, random and scattering corrections. For the assessment of tumor ^18^F-FDG uptake, all studies were exported and analysed using the PMOD software (PMOD Technologies Ltd., Adliswil, Switzerland). Regions of interest (ROIs) were drawn on coronal 1-mm-thick small-animal PET images on consecutive slices including the entire tumor. Finally, maximum standardized uptake value (SUV) was calculated for each tumor using the formula SUV = [tissue activity concentration (Bq/cm^3^)/injected dose (Bq)] × body weight (g).

### Lapatinib plus irradiation combination in vivo study

To assess the activity of lapatinib on A549 cells in response to irradiation, combination treatments (irradiation+lapatinib) were performed in nude mice. A549 tumor-bearing mice received a total irradiation dose of 16Gy (8 Gy/dose administered the second and third week after cell injection). For this experiment, mice were randomized into two groups: 1) X-ray irradiated alone and 2) the combination of lapatinib (100 mg/Kg) and irradiation at the indicated dose. Irradiation was performed with a Primus^® ^Linear Accelerator (Siemens AG, Erlangen, Germany) X-ray machine.

### Quantification of the circulating endothelial progenitors (CEPs)

To quantify the content of circulating endothelial progenitors (CEPs) in lapatinib-treated A549 xenografts by flow cytometry analysis, a volume of 100-200 μL peripheral blood was pre-incubated for 30 min at 4°C with 200 μL PBS-EDTA-BSA (phosphate 10 mM, 3% EDTA, 2% bovine serum albumin pH 7.4). Subsequently, samples were incubated in darkness for 30 min at 4°C with 7-aminoactinomycin-D (7AAD, Sigma-Aldrich), FITC-conjugated anti-mouse CD45, APC-conjugated anti-mouse CD117, and PE-conjugated anti-mouse Flk-1/KDR (the latter ones from BD Pharmingen). Cells were plotted according to forward scatter and side scatter profiles and gated to include only mononuclear cell events and to exclude cell doublets, platelets, dead cells/debris, microparticles and high side scatter events. The number of CEPs (CD45-CD117+VEGFR2+) were quantified and expressed as percentage (number of CEPs per hundred viable mononuclear cells).

### Immunohistochemistry for CD31 and quantification of tumor angiogenesis

A549 lung cancer tissues were fixed in 10% buffered formalin, embedded in paraffin, and sectioned (5 μm in thickness). Slides were stained with H&E and Masson Trichrome. For immunohistochemistry, slides were deparaffinized, incubated for 30 min with 3% H_2_O_2 _in methanol to quench the endogenous peroxidase activity and hydrated through graded alcohols. Antigen retrieval was carried out as follows: Slides were incubated with 50 μg/mL proteinase K for 30 min at 37°C and 20 min at room temperature. Tissues were then incubated with goat normal serum in buffer Tris- EDTA (TE) at 1:20 dilution for 30 min at room temperature. The anti-CD31 monoclonal antibody (BD Pharmingen) was diluted 1:25 in TE buffer and incubated overnight at 4°C. Slides were then incubated for 30 min at room temperature with a secondary rabbit anti-rat antibody at 1:200 dilution in TE buffer. Afterwards, slides were incubated for 30 min with the EnVision™ anti-rabbit detection system (Dako). Peroxidase activity was carried out with DAB (3,3'-diaminobenzidine, Dako). Finally, slides were counterstained with hematoxylin, dehydrated, and mounted with DPX. For quantifications, 30 random images (400×) per experimental group were captured with a microscope (Leica, Wetzlar, Germany) equipped with the Analysis™ software. CD31-positive vessels were quantified with the Axiovision 4.6 software (Zeiss). Measurements are given as relative area occupied by CD31-positive vessels with respect to the reference area.

### Statistical analysis

Unpaired two-tailed Student's *t *test was used to analyze comparisons between two groups. One-way ANOVA with Newman-Keuls multiple comparison Test was used to analyze vessel density data. Statistical differences were considered significant when p < 0.05.

## Results

### HER-2 gene amplification in A549 cells

**Figure 1:**
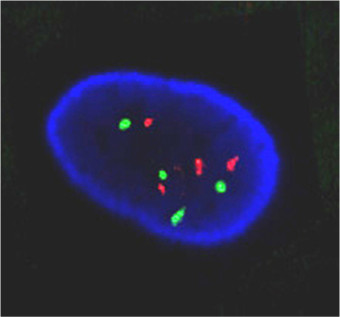
**FISH analysis showing 4 signals for HER-2 (red) and 4 signals for the chromosome 17 centromere (green) in A549 cells**.

Previous studies have shown that A549 cells harbor EGFR gene amplification [7], but the HER-2 status of these cells was unknown. We determined whether the HER-2 gene is also amplified in A549 cells. Using a DNA probe specific for HER-2 (red) and a DNA probe specific for the centromere of chromosome 17 (green), genetic analysis was carried out by Fluorescence *in situ *hybridization (FISH). Results showed that 13% of A549 cells contained normal copies of the HER-2 gene, as demonstrated by two green and two red signals. However, 21% of the nuclei exhibited HER-2 gene amplification as shown by four red HER-2 signals and two green centromeric signals. Most of the A549 nuclei were tetrasomic (four centromeric signals of chromosome 17 and 4-5 copies of HER-2); this accounted for 66% of all the nuclei of A549 cells. A representative picture of HER-2 FISH analysis is shown in Fig 1. Metaphases of A549 chromosomes were also analyzed confirming that the majority of cells exhibited tetrasomy for chromosome 17. We therefore conclude that similar to EGFR, A549 exhibits HER-2 gene amplification.

### Laptinib inhibits A549 lung cancer cell growth

**Figure 2:**
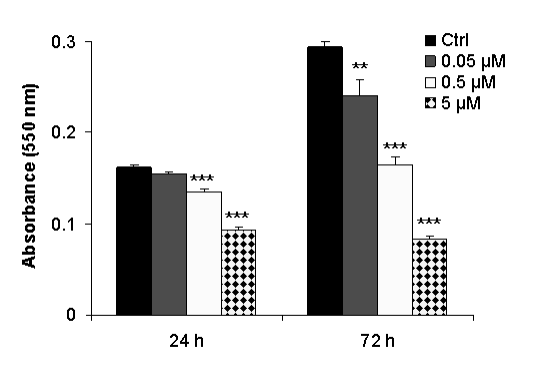
**Cytotoxicity of lapatinib in A549 cells exposed to different concentrations (0.05, 0.5 and 5 μM) of the drug, for 24 h and 72 h**. At the indicated time point, cell viability was measured by MTT, indicating that lapatinib significantly reduces A549 cell proliferation (**: *P *< 0.01; ***:*P *< 0.001).

**Figure 3:**
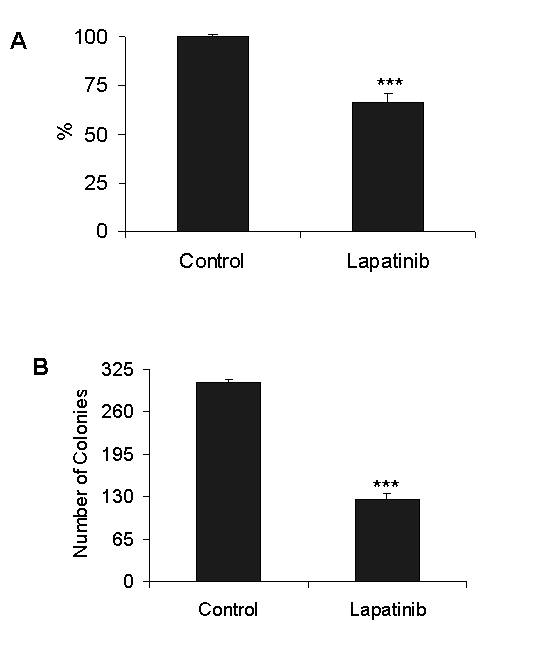
**Analysis of cell survival after 2 μM lapatinib treatment**. **A**. A549 cells exposed to 2 μM lapatinib for 24 h exhibited a reduced cell growth proliferation (*P *< 0.001); **B**. After 10 days of exposure to lapatinib, the number of colonies was dramatically abrogated (***: *P *< 0.0001).

A549 cells treated with lapatinib (0.05, 0.5 and 5 μM) for 24 h showed a dose dependent decrease in cell proliferation compared to controls. After 72 h of exposure, a strong reduction was observed (*P *< 0.0001) (Fig 2). For further experiments, we chose a 2 μM concentration, which produces 35% cell growth inhibition (Fig 3A). Clonogenic assays revealed that, whereas untreated cells gave rise to 305 ± 5 colonies, lapatinib-treated cells significantly reduced the colony formation ability to 127 ± 8 (*P *< 0.0001) (Fig 3B). These data show that Lapatinib inhibits the growth of the A549 lung cancer cell line.

### Lapatinib alters the cell cycle in A549 cells

**Figure 4:**
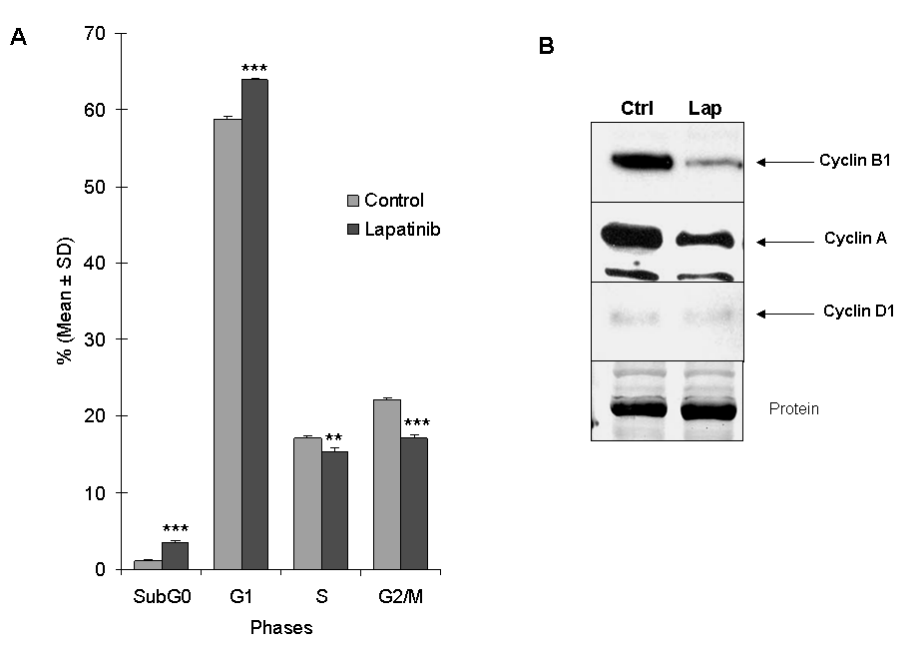
**Cell cycle study of A549 cells treated with lapatinib**. **A**. Lapatinib significantly alters cell cycle phases (G1 arrest and DNA synthesis reduction) analyzed by flow cytometry after propidium iodide staining (**: *P *< 0.01; ***: *P *< 0.001); **B**. Western blot showing the effect of lapatinib on the expression of the cell cycle regulators cyclins A, B1 and D1. In treated cells, the lower levels of cyclins A and B1, regulators of S and G2/M stages, respectively, corroborated the results described by cytometry. Levels of cyclin D1, a regulator of the G1 phase, were very low and remained unchanged upon treatment.

The antiproliferative activity of lapatinib in A549 cells prompted us to analyze the effect on the cell cycle. A549 cells treated with 2 μM lapatinib for 24 h resulted in a significant reduction in the S and G2/M phases (*P *< 0.006 and *P *< 0.0001, respectively). 17.0 ± 0.3% (mean ± SD) of untreated cells were in S phase and 22.1 ± 0.4% in G2/M phase, whereas 15.4 ± 0.4% in S phase and 17.1 ± 0.4% in G2/M phase were found for cells exposed to lapatinib (Fig 4A). In keeping with these results, the levels of cyclins A and B1, regulators of S and G2/M stages, respectively, were lower in lapatinib-treated cells compared to controls (Fig 4B). Treatment with lapatinib resulted in a significant increase in the percentage of cells in the G1 phase (from 58.9 ± 0.3% to 63.9 ± 0.2%; *P *< 0.0001). Basal levels of cyclin D1 (a regulator of the G1 phase) were very low in A549 cells, but no changes seemed to be produced upon lapatinib treatment (Fig 4B). Finally, administration of lapatinib caused a significant increase (3-fold) in the subG1 phase (suggestive of apoptosis): From 1.13 ± 0.08% to 3.37 ± 0.3%; *P *< 0.0006. Taken together, these data show that lapatinib causes cell cycle alterations with G1 arrest, DNA synthesis reduction and cell death induction, in A549 lung cancer cells.

### Alteration of the EGFR/HER-2 receptors and downstream signaling cascades by lapatinib results in apoptosis induction in A549 cells

**Figure 5:**
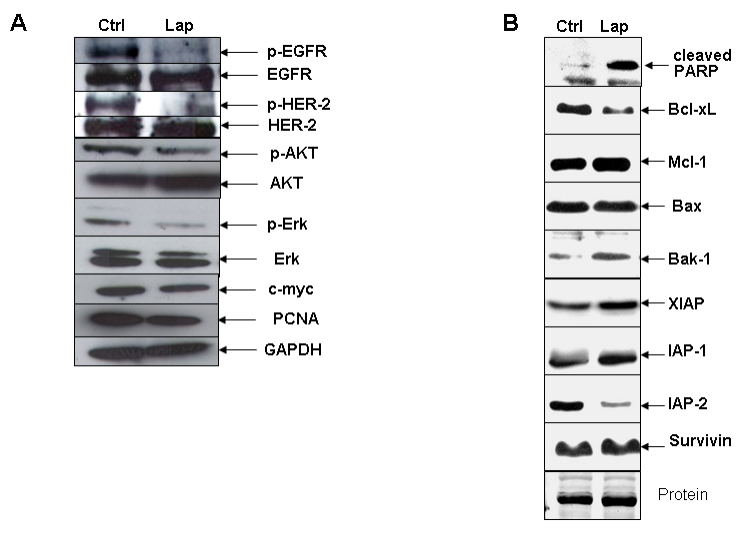
**Intracellular signaling changes induced by lapatinib in A549 cells, analyzed by western blot**. **A**. Immunoblots showing decreased levels of p-EGFR and p-HER-2 after stimulation with 100 ng/ml EGF and treatment with lapatinib. Downstream targets p-AKT, p-ERK1/2, c-Myc and PCNA were also reduced upon exposure to the drug. **B**. After lapatinib treatment, the proapoptotic protein Bak-1 was increased, the levels of the antiapoptotic proteins IAP-2 and Bcl-xL were reduced, and PARP was cleaved, thus demonstrating that the apoptotic pathway is switched on by this drug in A549 lung cancer cells.

To verify alterations in the EGFR/HER-2 receptors and downstream signaling pathways, we analyzed protein levels of p-EGFR, EGFR, p-HER-2, HER-2, p-ERK1/2, ERK1/2, p-AKT, AKT, c-myc, and PCNA (Fig 5A). As expected, lapatinib reduced levels of p-EGFR, p-HER-2, and p-ERK1/2 in A549 cells. Since studies in other tumor types have shown that the AKT pathway may also be perturbed by lapatinib, we analyzed p-AKT levels before and after treatment. Indeed, reduced levels in the phosphorylated form, but no changes in total AKT were found, after exposure to the drug. In addition, c-Myc and PCNA levels were also reduced (Fig 5A). Treatment with lapatinib resulted in an increase in cleaved PARP, which is a substrate for activated caspases (Fig 5B). Lapatinib reduced the levels of the two antiapoptotic proteins IAP-2 and Bcl-xL, and increased the levels of the proapoptotic protein Bak-1. However, no changes were found in the antiapoptotic proteins Mcl-1, IAP-1, XIAP, survivin and the proapoptotic protein Bax (Fig 5B). To confirm quantitatively the apoptotic induction, active caspase-3 was measured by flow cytometry. The following results were obtained: Twenty-four hours after treatment, 4.63 ± 0.77% and 4.59 ± 0.42% of the cells were positive when 2 μM or 5 μM were used (respectively), compared with 3.92 ± 0.22% for controls. Seventy two hours after the administration of the drug, the following values were found: 8.00 ± 0.18% for 2 μM, and 9.07 ± 0.22% for 5 μM, in comparison to 5.21 ± 0.18% for untreated control cells. These results indicate a proapoptotic effect induced in A549 lung cancer cells upon lapatinib treatment.

### Lapatinib activity in lung tumor xenografts

**Figure 6:**
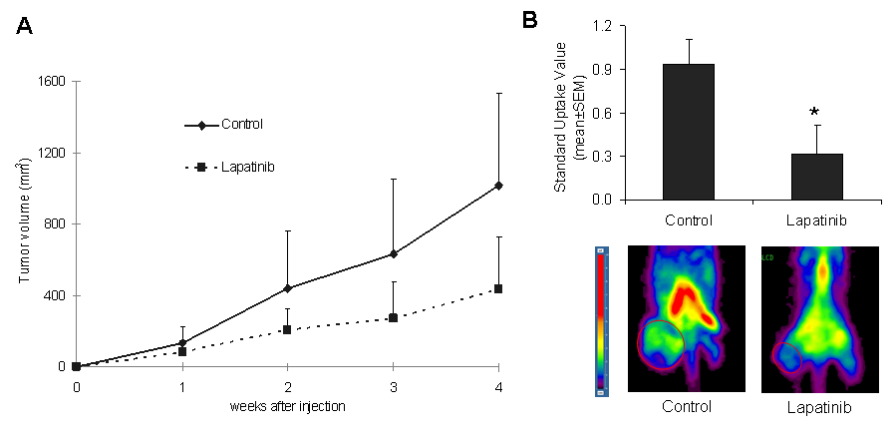
***In vivo *tumor growth assays**. **A**. After tumor implantation into immunocompromised nude mice, animals were treated with lapatinib for four weeks at the indicated concentration. Tumor volumes in treated mice were smaller than those found in controls; **B**. Lapatinib significantly reduced tumor metabolism (*P *= 0.037), which was shown by the *standardized glucose uptake values *(SUV) measured with micro-PET.

After 4 weeks of daily treatment of A549 tumor-bearing mice with lapatinib, tumor growth was reduced by more than 57% (on average) compared to controls (433 mm^3 ^versus 1015 mm^3^), although no statistical differences were reached, probably due to high variability of tumor growth in the control group (Fig 6A). However, measurement of tumor metabolism (^18^F-FDG uptake) with small animal PET analysis showed a significant reduction (*P *= 0.037) in mice treated with lapatinib compared to controls. SUV -- standardized uptake value (mean ± SEM)-- for controls was 0.94 ± 0.17, whereas the value for lapatinib-treated mice was 0.32 ± 0.20 (Fig 6B).

**Figure 7:**
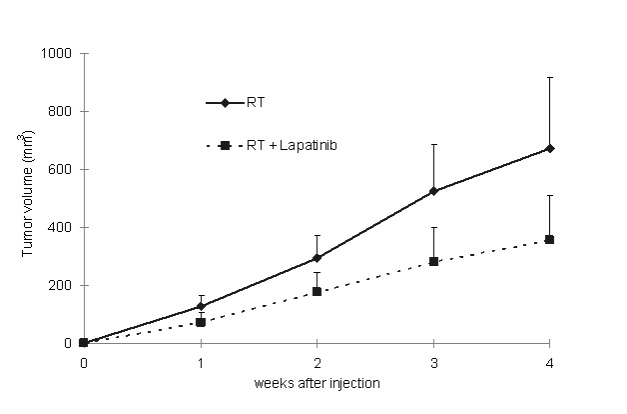
***In vivo *effect of radiotherapy alone or in combination with lapatinib, in A549 tumor-bearing mice**. Lapatinib reduced modestly tumor growth in irradiated animals compared to only irradiated mice.

Previous studies have shown that EGFR or HER-2 inhibition may potentiate the effect of radiation therapy [10]. We were particularly interested in testing if lapatinib can enhance the effect of radiotherapy in the A549 xenograft lung cancer model. Radiotherapy treatment (16 Gy) in combination with lapatinib reduced tumor volume with respect to radiotherapy alone by 48% (353 mm^3 ^versus 671 mm^3^) (Fig 7); however, no statistical differences were observed. Analysis of ^18^F-FDG uptake in tumors by PET showed that the metabolic activities in radiotherapy-treated and radiotherapy plus lapatinib-treated animals were similar (SUV = 0.56 ± 0.16 and 0.40 ± 0.13, respectively) (data not shown). Therefore, in the A549 xenograft lung cancer model, lapatinib does not enhance significantly the effect of radiotherapy.

### Lapatinib impairs angiogenesis and reduces circulating endothelial progenitors (CEPs) in A549 tumor-bearing mice

**Figure 8:**
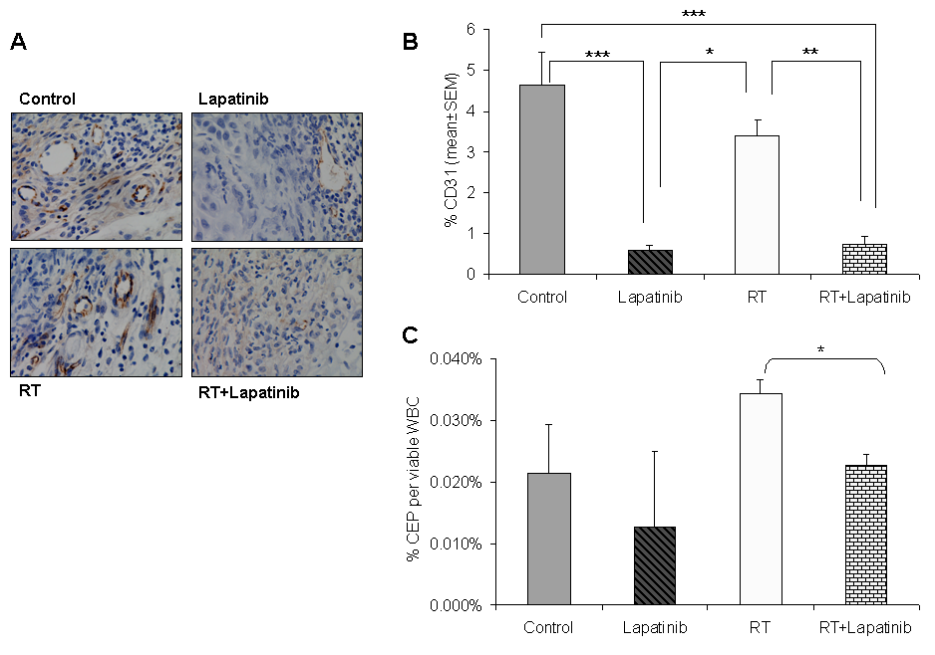
**Lapatinib alters angiogenesis and the number of circulating endothelial progenitors (CEPs) in mice xenotransplanted with A549 tumor cells**. **A**. Representative images of CD31-stained tumors from controls, lapatinib-treated, radiotherapy-treated and radiotherapy plus lapatinib-treated mice. **B**. Lapatinib dramatically reduced the CD31-positive area in the tumors (*: *P *< 0.05; **: *P *< 0.01; ***: *P *< 0.001); **C**. Quantification of CEPs in A549 tumor-bearing mice by flow cytometry from the peripheral blood. Lapatinib tended to reduce the number of CEPs compared to controls. Interestingly, it significantly diminished the number of CEPs (*P *= 0.0167) that were increased after radiotherapy treatment.

Since inhibition of EGFR and HER-2 has been shown to reduce angiogenesis through an indirect effect on VEGF production [11,12], we evaluated whether lapatinib interferes with tumor angiogenesis in the A549 model *in vivo*. Tumor angiogenesis (vessel density) was estimated by analyzing CD31-stained tumor sections. Lapatinib dramatically reduced vessel density compared to controls (0.59 ± 0.13 for lapatinib versus 4.6 ± 0.84 for controls; *P *< 0.0001) (Fig 8A and 8B). Inhibition of angiogenesis was also observed in irradiated mice treated with lapatinib compared to mice exposed to radiotherapy alone (0.75 ± 0.18 for lapatinib versus 3.39 ± 0.39 for radiotherapy; *P *< 0.01) or compared with the untreated controls (*P *< 0.0001) (Fig 8A and 8B). These results show that inhibition of angiogenesis may be an important mechanism *in vivo *elicited by Lapatinib.

We were further interested in elucidating the contribution of circulating endothelial progenitor cells (CEPs) to tumor angiogenesis. For this purpose, CEPs were measured in A549 tumor-bearing mice by flow cytometry from the peripheral blood. Although not statistically different, lapatinib treated-mice reduced the number of CEPs compared to untreated control mice (*P *= 0.584) (Fig 8C). In contrast, when mice were irradiated, the number of CEPs increased (*P *= 0.197) (Fig 8C) similar to what was previously described [13,14]. However, the combined treatment (radiation plus lapatinib) produced a significant reduction in the number of CEPs with respect to radiation alone (*P *= 0.0167) (Fig 8C). These results reinforce the idea that lapatinib impairs angiogenesis and reduces the number of CEPs in A549 lung tumor-bearing mice.

## Discussion

Although progress has been made in the management of advanced lung cancer, many challenges still remain. Chemotherapy is the primary treatment for advanced NSCLC patients. However, recent results suggest that no significant improvement in survival is likely to occur in those patients [15-17]. The overexpression of EGFR and HER-2, which has been observed in a significant number of lung cancer patients, offers an opportunity to block these tyrosine kinase receptors with targeted drugs [2,18]. The EGFR tyrosine kinase inhibitors erlotinib and gefitinib were approved by the US Food and Drug Administration (FDA) for the treatment of NSCLC. Although in randomized phase III clinical trials gefitinib was not associated with significant improvement in survival [19], its use has been proven clinically effective for patients with activating EGFR mutations [3]. Lapatinib is a novel dual EGFR and HER-2 tyrosine kinase inhibitor that is now approved by the FDA for treatment of metastatic breast cancers with overexpression of HER-2 receptors [20-22].

We used the A549 cell line model of NSCLC, which expresses EGFR and HER-2, to test the preclinical efficacy of lapatinib against lung cancer. Our results show that lapatinib inhibits the growth and increases apoptosis in these cells *in vitro*. More importantly, lapatinib inhibits A549 tumor activity and angiogenesis in a xenograft mouse model.

We have shown by FISH analysis that the HER-2 gene is amplified in A549 cells. This is consistent with previous studies that reported increased EGFR gene copy number in lung tumours [1,2]. Prediction of DNA alterations (gains or losses) to diverse genomic regions (chromosome 7, 11, 17 or 20 amplification and chromosomes 1, 2 or 9 deletions) in A549 cells have been recently associated with sensitivity to lapatinib [7]. Interestingly, in A549 cells, chromosomal gains were predicted in the region 17q12, where the HER-2 gene is located [7]. The A549 cell line might therefore constitute an appropriate preclinical model for testing the efficacy of lapatinib against NSCLC.

We demonstrate in this model that lapatinib-mediated blockade of both EGFR and HER-2 phosphorylation causes downstream signaling alteration upon drug administration. Similar to other EGFR inhibitors, such as erlotinib, lapatinib inhibited cell growth of A549 cells, and increased the proportion of cells in the G1 phase, while decreased those in the S and G2/M phases [23]. A possible reason for this cell cycle effect is the decrease in the protein levels of cyclins A and B1, which are regulators of S and G2/M phases, respectively. Lapatinib-induced inhibition of cyclins A and B1 likely slows down progression through the S and G2/M cell cycle phases, contrasting with the result showing no change in cyclin D1, a mediator of the G1 phase. This very same phenomenon has been observed with erlotinib [23].

We found that lapatinib blocks ERK1/2 phophorylation in A549 lung cells, as previously described in lapatinib-treated breast cancer cells [24]. Furthermore, p-ERK1/2 downregulation is followed by a downstream reduction of c-Myc, which might contribute to the aforementioned G1 arrest. A recent work also demonstrated that c-Myc is a target of lapatinib in gastric cancer cell lines [25]. In addition, these data are consistent with other reports demonstrating that cyclin A is critical for c-Myc-modulated cell cycle progression [26]. Therefore, lapatinib inhibition of cyclin A may subsequently abrogate c-Myc and, in turn, induce G1 phase arrest in A549 cells.

An important feature of anti-cancer agents is the ability to trigger apoptotic cell death. Our results show that treatment of A549 cells with lapatinib causes apoptosis, as determined by an elevated proportion of cells in the sub-G1 cell cycle phase, and increased cleaved PARP and active caspase-3. Moreover, lapatinib decreased levels of the anti-apoptotic proteins Bcl-xL and IAP-2. Bcl-xL is a member of the Bcl-2 family that acts on the mitochondrial membrane to prevent release of caspase activators such as cytochrome-C [27]. Overexpression of inhibitor of apoptosis (IAP) family members (IAP-2 among them) can also inhibit caspase activation, block apoptosis, and increase drug resistance [28]. Therefore, the ability of lapatinib to reduce the levels of Bcl-xL and IAP-2 should increase mitochondria outer membrane permeabilization (MOMP), release cytochrome-C, and induce apoptosis. Results presented here also show increased Bak-1 (a proapoptotic member of the Bcl-2 family) levels, which are required along with Bax to increase MOMP and apoptosis. The correlation between lapatinib-induced cell death, Bak activation and Bcl-xL downregulation has been described as well in colon cancer cells [29].

The growth-inhibitory effects of lapatinib were also evaluated *in vivo*. In the A549 xenograft model, this drug reduced tumor growth, and glucose uptake (indicating decreased metabolism and proliferative activity of tumor cells). PET analysis has also been used in NSCLC patients to monitor the response to the EGFR tyrosine kinase inhibitor gefitinib [30]. *In vivo *experiments using lapatinib in combination with radiotherapy showed no therapeutical benefit as compared to the use of each therapy alone in our study. Therefore, at least in these experimental settings, lapatinib does not enhance the therapeutic effect of radiotherapy. Randomized trials using lapatinib were recently initiated in patients with locally advanced squamous cell carcinoma of head and neck [10] and NSCLC [4]. Results from these studies and from other preclinical models will determine whether the use of lapatinib alone or in combination with other therapeutical agents may result in clinical benefit.

*In vivo *preclinical experiments using EGFR and VEGFR inhibitors in colon cancer models reported several important findings: First, both receptors were present in tumor-associated mouse endothelial cells; and second, targeting both receptors with the tyrosine kinase inhibitor AEE788 reduced tumor growth and caused apoptosis in both tumor and endothelial cells [31]. We have found in the present study that treatment with lapatinib decreases dramatically tumor angiogenesis (by >80%) as compared to controls. This result suggest that blockade of angiogenesis may be one critical *in vivo *mechanism elicited by lapatinib. It is possible that inhibition of EGFR downstream signaling reduces the expression of angiogenic factors (such as VEGF, IL-8, etc.) through indirect mechanisms. Interestingly, Olaussen et al. [32] have recently demonstrated that lapatinib inhibits VEGFR1 phosphorylation by >70% in A549 cells. Although such an effect has not been shown in endothelial cells, one could assume that VEGFR1 phosphorylation blockade would have a direct antiangiogenic effect. These issues should be addressed in future studies.

Both peritumoral vessels and circulating bone-marrow-derived endothelial progenitors (CEPs) contribute to tumor angiogenesis [33]. In addition, CEPs are significantly increased in NSCLC patients and are associated with poor prognosis [34]. Our results show that lapatinib reduces modestly the number of CEPs. Radiotherapy caused an increase in CEPs in our *in vivo *model, similar to that previously described in response to stress or therapy, including radiation [13,14,35-37]. Interestingly, after tumor irradiation and lapatinib administration, the number of CEPs was significantly reduced. Therefore, a potential mechanistic function of lapatinib could be the inhibition of endothelial cell recruitment to the tumor.

## Conclusion

Our results show that lapatinib has antitumor activity *in vitro *and *in vivo *against lung cancer, but does not act as an enhancer of radiotherapy. Further studies will be required to assess whether lapatinib alone or in combination with chemotherapy may be clinically relevant to treat human lung cancer.

## Conflict of interests

The authors declare that they have no competing interests.

## Authors' contributions

RD and PAN participated in the design and writing of the manuscript, and undertook the vast majority of the experimentation and analysis of the data. RP, CPE, and IM conducted some of the western blots and helped in the analysis and interpretation of the results of the study. MR performed the immunohistochemical analysis and helped in other experimental and analytical procedures of the manuscript. RC contributed with the *in vivo *experimentation and study design. MC and IP carried out microPET analyses. JAD participated in the study design and paper editing. AC contributed in the study design, monitoring of the experimentation and writing the paper. All authors read and approved the manuscript.

## Pre-publication history

The pre-publication history for this paper can be accessed here:

http://www.biomedcentral.com/1471-2407/10/188/prepub
